# Coculture of hWJMSCs and pACs in Oriented Scaffold Enhances Hyaline Cartilage Regeneration *In Vitro*

**DOI:** 10.1155/2019/5130152

**Published:** 2019-02-07

**Authors:** Yu Zhang, Shuyun Liu, Weimin Guo, Chunxiang Hao, Mingjie Wang, Xu Li, Xueliang Zhang, Mingxue Chen, Zehao Wang, Xiang Sui, Jiang Peng, Yu Wang, Shibi Lu, Quanyi Guo

**Affiliations:** ^1^Institute of Orthopaedics, Chinese PLA General Hospital, Beijing Key Lab of Regenerative Medicine in Orthopaedics, Key Laboratory of Musculoskeletal Trauma & War Injuries, PLA, 28 Fuxing Road, Haidian District, Beijing 100853, China; ^2^Department of Sports Medicine and Adult Reconstructive Surgery, Nanjing Drum Tower Hospital, The Affiliated Hospital of Nanjing University Medical School, 321 Zhongshan Road, Gulou District, Nanjing 210008, China; ^3^Institute of Anesthesia, Chinese PLA General Hospital, 28 Fuxing Road, Haidian District, Beijing 100853, China; ^4^School of Medicine, Nankai University, Tianjin 300071, China; ^5^Shanxi Traditional Chinese, No. 46 Binzhou West Street, Yingze District, Taiyuan 030001, China

## Abstract

Seed cells of articular cartilage tissue engineering face many obstacles in their application because of the dedifferentiation of chondrocytes or unstable chondrogenic differentiation status of pluripotent stem cells. To overcome mentioned dilemmas, a simulation of the articular cartilage microenvironment was constructed by primary articular cartilage cells (pACs) and acellular cartilage extracellular matrix- (ACECM-) oriented scaffold cocultured with human umbilical cord Wharton's jelly-derived mesenchymal stem cells (hWJMSCs) *in vitro.* The coculture groups showed more affluent cartilage special matrix ingredients including collagen II and aggrecan based on the results of histological staining and western blotting and cut down as many pACs as possible. The RT-PCR and cell viability experiments also demonstrated that hWJMSCs were successfully induced to differentiate into chondrocytes when cultured in the simulated cartilage microenvironment, as confirmed by the significant upregulation of collagen II and aggrecan, while the cell proliferation activity of pACs was significantly improved by cell-cell interactions. Therefore, compared with monoculture and chondrogenic induction of inducers, coculture providing a simulated native articular microenvironment was a potential and temperate way to regulate the biological behaviors of pACs and hWJMSCs to regenerate the hyaline articular cartilage.

## 1. Background

The articular cartilage plays an important role in load distribution, reducing friction, and pain-free movement [1]. However, an injured articular cartilage is rarely self-healing because of limited in situ cell migration in dense cartilage tissue and the lack of nutrient supplies, thus osteoarthritis will develop over time [2, 3]. Tissue engineering combined with seed cells, biomaterials, and cytokines is one of the most promising ways to repair and regenerate the articular cartilage and avoid the progress of osteoarthritis. Seed cells, the core elements of tissue engineering, determine the regenerative tissue properties and function. Mesenchymal stem cells (MSCs) derived from different connective tissue and articular cartilage cells (ACs) are the main sources for articular cartilage tissue engineering [4, 5].

In general, in order to construct the hyaline cartilage, the MSCs need to differentiate into chondrocytes by adding chondrogenic inducers or transferring chondrogenic genes, such as TGF-*β* [6, 7]. After cultivation in the presence of chondrogenic inducers *in vitro* for a certain amount of time, MSCs were observed to differentiate into chondrocytes; cartilage-specific genes and proteins were upregulated, such as collagen II and aggrecan. However, the induced chondrocytes hardly stayed in this special status because of inadequate induction leading to fibrocartilage or hypertrophic chondrocytes resulting in overcalcified cartilage instead of hyaline articular cartilage [8–11]. It is difficult to measure (i) the optimal dose of chondrogenic inducer, (ii) the optimal cultivation time, and (iii) the optimal degree of upregulation of chondrocyte-associated genes. Therefore, the *in vivo* generation of hyaline articular cartilage containing stable differentiated chondrocytes remains a challenge. Chondrocytes have also been identified as potential seed cells for articular cartilage tissue engineering. Autologous chondrocyte implantation (ACI) was widely studied and applied in clinical settings to treat damaged articular cartilage [8, 12]. Primary articular cartilage cells (pACs) have to be isolated from the articular cartilage tissue and cultured *in vitro* to obtain sufficient quantities to construct tissue engineering cartilage. Brittberg and his group first published ACI technology to repair deep cartilage defects in the femorotibial articular surface of the knee joint in 23 patients and believed that cultured autologous chondrocytes can be used in clinic [13]. Peterson et al. found hyaline cartilage formation from ACI in several patients based on safranin “O” staining results [14]. Furthermore, the superiority of cartilage regeneration by matrix-applied (I/III collagen membrane) characterized autologous cultured chondrocytes (MACI) over micro fracturing has been demonstrated by Saris and his group using a two-year follow-up of a prospective randomized trial [15, 16]. Many studies reported that chondrocytes underwent dedifferentiation after many passages, as it was observed that the cells lost their native hyaline cartilage phenotype. Passage-dependent dedifferentiation of chondrocytes was first published by Dell'Accio et al. who found that the finite capacity to form stable cartilage *in vivo* using a nude mouse model would be lost through passaging [17]. This was also an essential reason behind the inferior cartilage repair *in vivo* [18].

In recent years, to solve the problems of the unstable status of MSCs induced towards chondrogenic differentiation and the insufficient cell resources and dedifferentiation of articular cartilage cells, coculture of MSCs and chondrocytes has been suggested to effectively overcome the mentioned obstacles and acquire hyaline cartilage repair and regeneration. In the coculture system, a microenvironment greatly influences their biological behavior (including proliferation, differentiation, and phenotype maintenance) by biostimulation of cell-cell, cell-extracellular matrix, and cell-cytokine interactions [19–21]. Some reports demonstrated that companion cells (such as MSCs) secrete trophic or cell-cell communication factors to promote proliferation and delay dedifferentiation of cartilage cells while chondrocytes can induce the differentiation of multiple potentially different MSCs to chondrocytes without the need to add an inducer [11, 22]. Besides, coculturing reduces the required amount of cartilage tissue, decreases the *in vitro* culture time, and lowers the degree of chondrocyte degeneration [18]. However, some studies showed that coculture only exerted unidirectional trophic effect of MSCs on chondrocytes [23, 24]. Those differences probably were attributed to the complex microenvironment that (i) comprises a milieu of biochemical, biomechanical, and bioelectrical signals derived from the surrounding cells, the extracellular matrix (ECM), and soluble factors and (ii) regulates cell metabolism and function.

In our previous research, hWJMSCs evoked a very low immune response and repaired full-thickness articular cartilage defects in a New Zealand white rabbit model [25–27]. Some research showed that hWJMSCs possessed better chondrogenic differentiation capacity compared with BMSCs [28–30]. ACECM-oriented scaffold was preciously manufactured in our laboratory, retained an amount of native articular cartilage extracellular matrix components and oriented structure, and showed good biocompatibility and safety in animal models [31–33]. In this study, we attempt to reproduce the in situ articular cartilage microenvironment using unpassaged pACs, possessing native phenotype and an ACECM-oriented scaffold, which presumably stimulates a partially damaged microenvironment for cocultured hWJMSCs to construct tissue engineering hyaline cartilage by inducing chondrogenic differentiation. The changes in the biological behavior of the cells were detected *in vitro*.

## 2. Coculture Studies *In Vitro*

### 2.1. Isolation, Culturing, and Identification of hWJMSCs and pACs

The acquisition of fresh human umbilical cords and implementation of animal experiments were consented and approved by the Chinese PLA General Hospital (Beijing, China) Ethics Committee. The details of hWJMSC isolation were previously described using micromass mesenchymal tissue placed in a culture dish with DMEM/F12 (10% FBS) [25]. The cells were collected after the third passage using 0.05% Trypsin-EDTA (Invitrogen) and identified by flow cytometry. Cells were stained with anti-human antibodies against the following cell surface markers: CD73-PE, CD90-PE, CD105-PE, CD34-PE, CD45-Percp, and HLA-DR-FITC (10 *μ*L).

The pACs were isolated from the articular cartilage tissue in femoral condyles of healthy adult goats under aseptic and painless conditions using type II collagenase after cutting into small pieces, which was approved by the Institutional Animal Care and Use Committee of the Chinese PLA General Hospital. When the cells reached about 70% confluency culturing in DMEM/F12 (10% FBS) medium, types I and II collagen immunofluorescence staining was performed to identify the characteristics of the cells derived from the articular cartilage tissue. The primary antibodies against collagen I (ab23446, Abcam, England), collagen II (ab34712, Abcam, England), secondary antibodies of donkey anti-mouse IgG H&L (Alexa Fluor® 594), and Donkey anti-rabbit IgG H&L (Alexa Fluor® 488) were used in this assay.

### 2.2. Cell Scaffold Complex

hWJMSCs and pACs were mixed in suspension (10^7^cells/100 *μ*L) at the following ratios: 100 : 0, 75 : 25, 50 : 50, 25 : 75, and 0 : 100 before dropping into an ACECM-oriented scaffold to construct the cell scaffold complex. The production of the ACECM-oriented scaffold was described previously [31]. The specifications of the scaffold are 1 mm thickness and 6.5 mm diameter. Cell scaffold complexes were cultured in DMEM/F12 (10% FBS) at 5% CO_2_, 37°C.

### 2.3. Gross Morphology and Histology Evaluation

After coculturing for 1 week and 3 weeks, the evaluations of the gross morphology (color and shape) of the cell scaffold complexes were performed. Then, the engineering tissue of cell scaffold complexes was embedded in optimum cutting temperature compound (OCT, CellPath, England) and cut into slices of 8 *μ*m. All the slices were fixed in acetone for 15 minutes, rinsed under running water, and then stained with H&E and safranin “O” to evaluate the cell distribution and structural features including the amount of proteoglycan which is a marker of chondrocyte matrix production, respectively. To determine the cartilage characteristics of the cell scaffold complexes, immunofluorescence experiments were conducted. The sections were stained with primary antibodies (Abcam, England) against collagen I (1 : 100; ab23446) and collagen II (1 : 100; ab34712) at 4°C overnight. After 60 minutes of incubation with secondary antibodies and 3 minutes of counterstaining of nuclei with Hoechst 33258 (Molecular Probes, Eugene, OR), images were taken using an Olympus fluorescence microscope.

### 2.4. Western Blot Analysis

Total protein extracts of cells scaffold complexes from each group were prepared for western blot analysis using the BCA protein assay kit (Hyclone Pierce, USA). The same amounts of cell lysates were loaded for SDS-polyacrylamide gel electrophoresis (SDS-PAGE) and transferred to polyvinylidene fluoride (PVDF) membrane. Immunoblotting was coupled with fluorescent signal detection using the ECL western blotting substrate kit (BioVision). The integral optical density was counted by Quantity-One software of a Gel-Doc imaging system. The following antibodies were used for this study: anti-aggrecan (ab3773, 1 : 100; Abcam); anti-collagen I (ab23446, 1 : 100; Abcam); anti-collagen II (ab34712, 1 : 100; Abcam); and anti-beta Actin (ab8227, 1 : 100; Abcam).

### 2.5. Cell Viability and Proliferation Assay

After coculturing for 1 day and 3 days, the cell viability of the cell scaffold complexes was assessed using Fluorescein diacetate (FDA)/propidium iodide (PI). The cell scaffold complexes were stained with FDA (5 *μ*g/mL) and PI (5 *μ*g/mL) for 5 minutes then rinsed three times with PBS. The samples were visualized under a confocal microscope (Olympus FV1200; Japan).

In order to observe the effect of the coculture system on the proliferation of hWJMSCs and pACs, both cell types were labeled with lentivirus loaded with green and red fluorescent genes. Cells were transfected at 70-80% confluency in six-well plates. hWJMSCs and pACs were transduced using 1 mL of purified lentivirus, 1 mL DMEM/F-12 (10%FBS), and 2 *μ*L of 5 *μ*g/*μ*L Polybrene at 37°C in a 5% CO_2_ atmosphere for 5 hours. The transduced liquid was then replaced with fresh cell culture medium to culture cells for 24 hours. Medium containing puromycin was applied 48 hours posttransduction. Stable clones were generated and cultured until sufficient quantities were obtained before implantation in scaffolds. The same total number of green-labeled hWJMSCs, red-labeled pACs, and a 50 : 50 mixture was seeded onto scaffolds of identical size. After culturing for 1 day and 5 days, the area of interest (AOI) of red and green fluorescence intensity was examined and quantified by laser scanning confocal microscopy and image-pro plus 6.0 based on the same parameters. It indirectly indicated the amount of hWJMSCs and pACs and we thus analyzed the effect of coculturing on the proliferation rate. The relative growth ratio based on fluorescence intensity was acquired as follows: (AOI (day 5) - AOI (day 1))/AOI (day 1).

### 2.6. Real-Time PCR Assay

The RT-PCR assay was used to analyze the changes in gene expression levels for pACs and hWJMSCs after coculturing them on ACECM-oriented scaffold for three weeks. Total RNA was extracted from three samples of each group using TRIzol reagent. RNA quality from each sample was ensured by the A260/280 absorbance ratio. The isolated RNA was reverse-transcribed into a single strand of cDNA using ReverTra Ace® qPCR RT Master Mix (Toyobo, Japan). All oligonucleotide primer sets were designed based upon the published mRNA sequence. The real-time PCR was performed by using the Step One RT-PCR (Applied Biosystems, USA) with SYBR Green Real-time PCR Master Mix-Plus (Toyobo, Japan). cDNA template 1 *μ*L was used for real-time PCR in a final volume of 20 *μ*L. cDNA was amplified according to the following conditions: 95°C for 15 s, 55°C for 30 s, and 72°C for 30 s at 40 amplification cycles. Fluorescence changes were monitored with SYBR Green after every cycle. The mRNA expression levels relative to GAPDH were determined using the 2-^ΔΔ^CT method. The statistical analysis of gene expression of hWJMSCs and pACs in the CC groups was carried out based on the single hWJMSC and pAC reference groups. The following primers used in this study are listed in Table 1 (Supplementary Materials).

### 2.7. Statistical Analysis

Data was analyzed using SPSS 18.0 (SPSS Inc., Chicago, IL, USA). The quantified data are described as means ± standard deviation. One-way analysis of variance (ANOVA) with the Student Newman-Keuls (SNK) was used to determine the significant differences between the two groups from *n* = 3. A value of *p* < 0.05 was considered to indicate a significant difference.

## 3. Results

### 3.1. Coculture Studies *In Vitro*

#### 3.1.1. Characteristics of hWJMSCs and pACs

**Figure 1:**
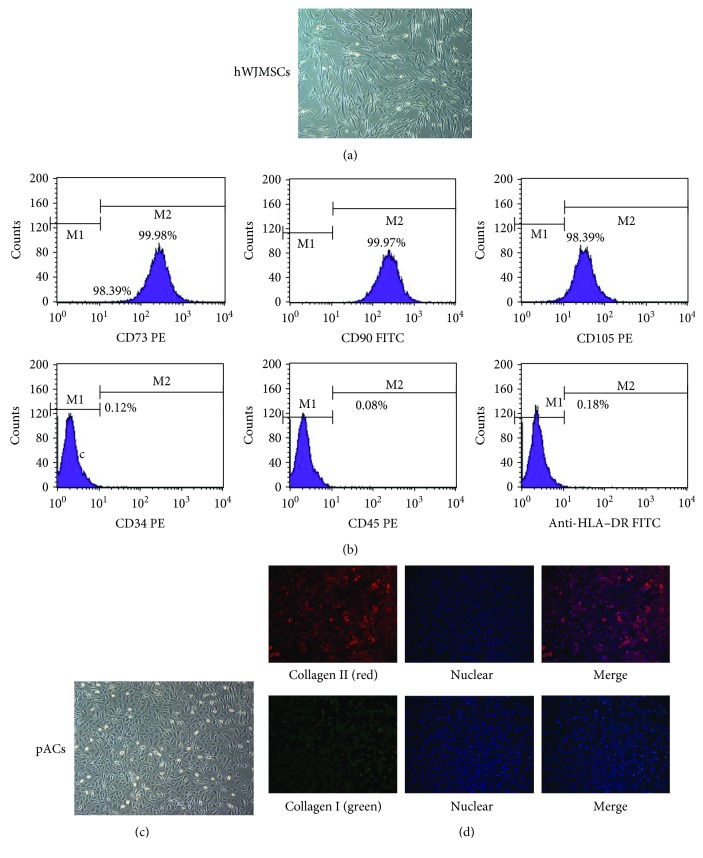
hWJMSCs and pACs after three passages satisfied the characters of MSCs and hyaline cartilage, respectively. (a) hWJMSCs revealed spindles and a fibroblast-like shape, ×40. (b) Expression of surface markers on hWJMSCs by flow cytometry revealed that CD73, CD90, and CD105 were positive, while CD34, CD45, and HLA-DR were negative. (c) pAC morphology showed a polygonous or round-shaped form, ×40. (d) Immunofluorescence staining of collagen II (red) of pACs was much more intense than that of collagen I (green).

Microscopic analysis showed that hWJMSCs exhibited a predominantly fibroblast-like morphology with spindle cells, suggesting that the cells displayed a typical mesenchymal morphology (Figure 1(a)), while the pACs presented polyphology or round-like morphology and some of them were in the phase of division. The flow cytometry analysis of specific MSC markers showed that hWJMSCs highly expressed CD73, CD90, and CD105 (≥95%) and lowly expressed the hematopoietic cell markers CD34, CD45, and HLA-DR (≤2%) (Figure 1(b)), which demonstrated that our batches of stem cells were very pure and only contained mesenchymal cells [28].

Under an inverted microscope, the primary cells isolated from the articular cartilage tissue grew and divided, uniformly shaped like a diamond or polygon (Figure 1(c)). In our immunofluorescence staining experiments, we observed that pACs were surrounded by dense collagen II (the strong red fluorescence), while collagen I only slightly coated the surface of pACs (green fluorescence). This evidently illustrated that the unpassaged pACs displayed a native hyaline cartilage cell phenotype, expressing mainly collagen II and less collagen I (Figure 1(d)).

### 3.2. Gross Morphology and Histology Evaluation of the Cell Scaffold Complex

**Figure 2:**
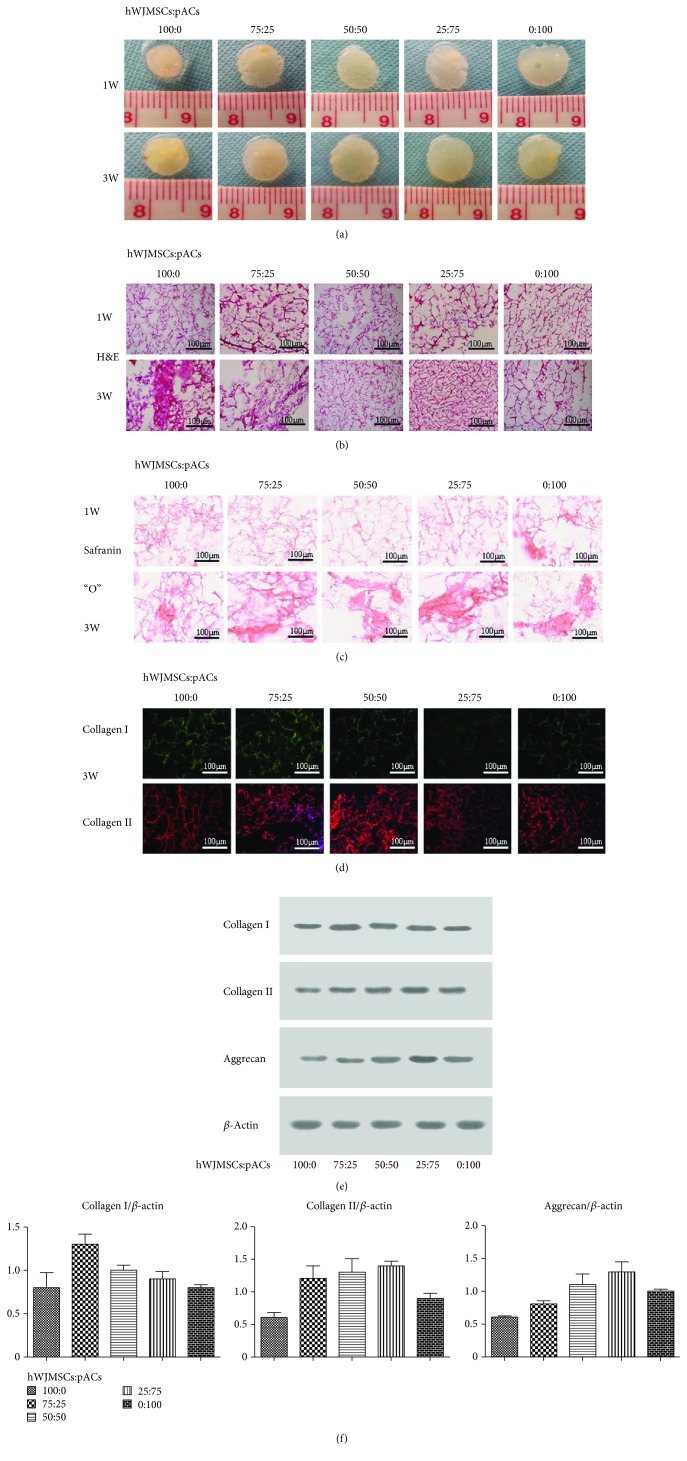
Coculture groups secreted abundant glycosaminoglycan and collagen II. (a) The gross morphology of hWJMSCs : pACs (100 : 0, 75 : 25, 50 : 50, 25 : 75, and 0 : 100). (b) H&E staining results. (c) Safranin “O” staining results. (d) The results of collagen I and collagen II immunofluorescence staining; 1 W: 1 week; 3 W: 3 weeks. (e) After coculturing for 3 weeks, western blotting analysis of collagen I, collagen II, and aggrecan; *β*-actin used as an internal reference protein. (f) The mean grey value of collagen I, collagen II, and aggrecan relative to that of *β*-actin. The results were the means ± SD; *n* = 3, ^∗^ *p* < 0.05.

After cultivation for 1 week and 3 weeks, the morphology showed the cell scaffold complexes consisted of soft transparent tissue, and especially at the third week, the pores were filled with extracellular matrix and cells. The density of the tissue in the 100 : 0 group was higher than that in any other group regardless of the time point, but the appearance of the tissue in the 100 : 0 group was not as transparent as in other groups (Figure 2(a)).

In our H&E staining experiments, we observed that the seed cells were evenly distributed over the oriented scaffold and adhered to the wall, which shows that the cell suspension dropping was an effective way to seed cells into the scaffold with high biocompatibility. Cells were denser at 3 weeks than at 1 week in their own groups, and the 100 : 0 group showed the densest cell distribution at 3 weeks (Figure 2(b)). As shown by safranin “O” staining, other than the 0 : 100 group, no group showed a significantly positive area at 1 week. At 3 weeks, particularly in the coculture groups, the safranin “O” staining was evidently stronger than that of the same groups at 1 week. Clearly, seed cells secreted much more glycosaminoglycan extracellular matrix in the coculture groups than in the 100 : 0 and 0 : 100 groups after culturing for 3 weeks and the dominance of the cocultures emerged at 3 weeks (Figure 2(c)).

The ACECM-oriented scaffold was made from extracellular matrix that was rich in collagen II as well as some collagen I, which contributed to the positive results in every group. However, at 3 weeks the obvious difference in coculture groups was that collagen II immunofluorescence staining was still obviously positive compared with collagen I immunofluorescence staining in the same groups, with a smaller porosity and bolder outline. Seed cells after coculturing for three weeks mainly secreted collagen II; little collagen I expression was found (Figure 2(d)).

### 3.3. Western Blot Analysis

From the western blot results, the amount of collagen I was highest in the 75 : 25 group, at 1.3 ± 0.2 grey scale grades, and the 100 : 0 and 0 : 100 groups showed the lowest collagen I levels at a mean of 0.8 on the grey scale grade. The groups showed a different fold increase (or decrease) of cell scaffold complex aggrecan levels, namely, from high to low, 25 : 75 (1.4-fold), 50 : 50 (1.3-fold), 75 : 25 (1.2-fold), and 100 : 0 (0.6-fold). There was no significant difference in the amount of collagen II among the 25 : 75, 50 : 50, and 75 : 25 groups (Figures 2(e) and 2(f)).

### 3.4. Cell Viability Staining and Proliferation in ACECM-Oriented Scaffold

**Figure 3:**
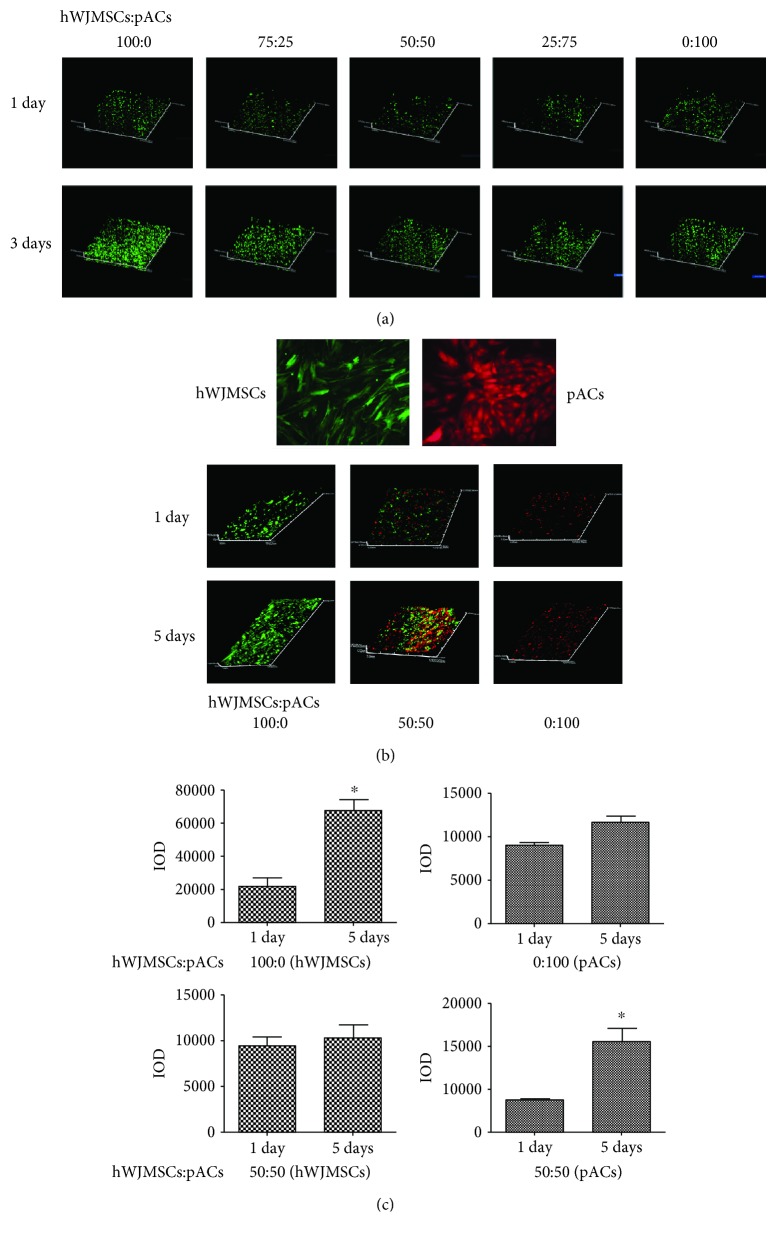
ACECM-oriented scaffold has good biocompatibility and the proliferation activity of pACs was significantly enhanced. (a) Dead/live staining of cell scaffold complexes using FDA/PI assay after coculturing for 1 day and 3 days by laser scanning confocal microscope, ×40; red color represents a dead cell and green color represents a living cell. (b) Green fluorescent lentivirus-labeled hWJMSCs, red fluorescent lentivirus-labeled pACs, laser scanning microscope, ×200; the proliferation assay was performed in three different groups (hWJMSCs : pACs), 100 : 0, 50 : 50, and 0 : 100, after coculturing for 1 day and 5 days; laser scanning confocal microscope, ×40. (c) 100 : 0 (hWJMSCs), IOD of hWJMSCs in the 100 : 0 group at 1 day and 5 days; 100 : 0 (pACs), IOD of pACs in the 100 : 0 group at 1 day and 5 days; 50 : 50 (hWJMSCs), IOD of hWJMSCs in the 50 : 50 group at 1 day and 5 days; 50 : 50 (pACs), IOD of pACs in the 50 : 50 group at 1 day and 5 days. The results are shown as means ± SD; *n* = 3, ^∗^ *p* < 0.05.

The cell viability was checked using the FDA/PI assay after culturing for 1 day and 3 days on the ACECM-oriented scaffold. Our laser scanning confocal microscopy results demonstrate that the green fluorescence cells (living cells) were much more abundant and showed a longitudinal distribution in the oriented scaffold, and red fluorescence cells (dead cells) showed no significant difference among the groups at 1 day or 3 days (Figure 3(a)). The ACECM-oriented scaffold and coculture model of the two kinds of seed cells did not illustrate a noticeably negative influence on the seed cell viability, while stimulating cell growth.

Further investigation of the influence of hWJMSCs and pACs on each other's proliferation during coculturing was very interesting. The IOD SUM of green and red fluorescence in three-dimensional cell scaffold complexes was counted and recorded (Figure 3(b)). Compared with hWJMSC single cultures (from 1 day to 5 days), the relative radio of green fluorescence of hWJMSCs was obviously lower after coculturing for 5 days, sliding from 2.09 to 0.09. However, the relative radio of red fluorescent lentivirus-labeled pACs in the coculture group (1.84) was higher than that in the single culture group (0.29). In brief, compared with the single culture, the proliferation of pACs was significantly improved and that of hWJMSCs was surprisingly lower after coculturing for 5 days. pACs and hWJMSCs cocultured with each other cause different effects on individual proliferation (Figure 3(c)).

### 3.5. Real-Time PCR Assay

**Figure 4:**
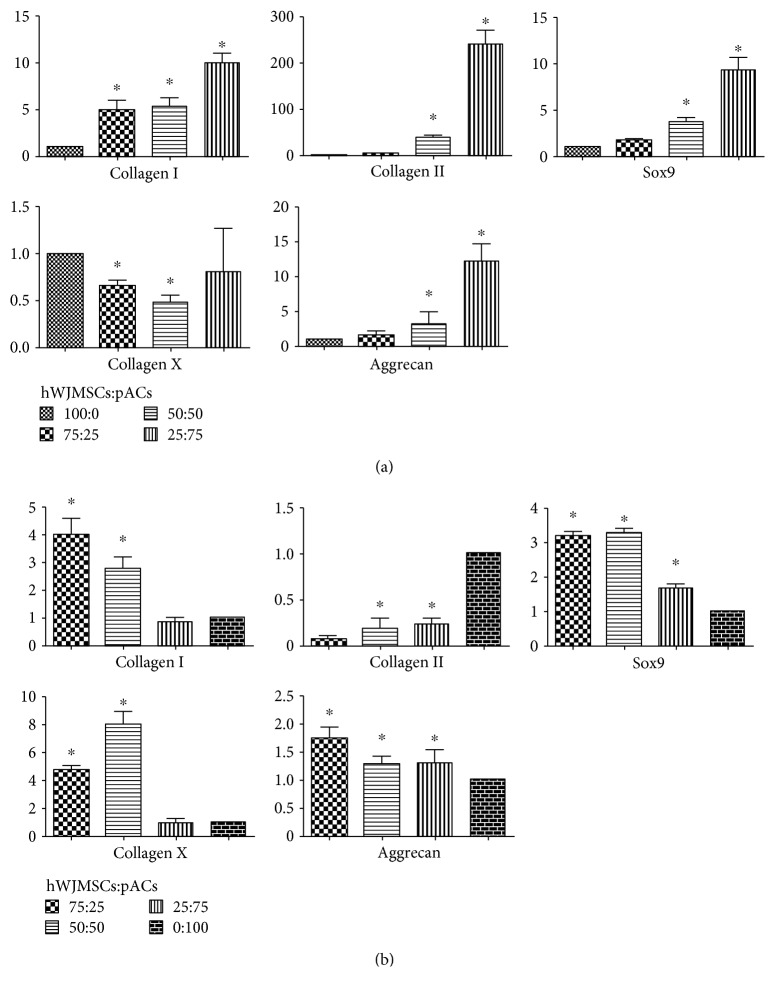
The chondrocyte-specific genes of hWJMSCs were upregulated. (a) The expression values of genes collagen I, collagen II, collagen X, Sox9, and aggrecan of hWJMSCs in the 75 : 25, 50 : 50, and 25 : 75 groups relative to the 100 : 0 group after coculturing for three weeks as measured by RT-PCR. (b) The expression values of genes collagen I, collagen II, collagen X, Sox9, and aggrecan of pACs in the 75 : 25, 50 : 50, and 25 : 75 groups relative to the 0 : 100 group after coculturing for three weeks. The results are shown as means ± SD; *n* = 3, ^∗^ *p* < 0.05.

Relative to the single hWJMSC (100 : 0) group, hWJMSCs of coculture groups (75 : 25, 50 : 50, and 25 : 75) revealed upregulated levels of collagen I, collagen II, Sox9, and aggrecan and downregulated levels of collagen X. The expression of the hyaline cartilage associated with gene collagen II increased with the higher ratio of pACs in coculture groups; the 50 : 50 group showed an upregulation with 39.00 ± 7.80 and the 25 : 75 group an upregulation with 239.35 ± 50.57. Sox9, a critical regulatory transcription factor for the expression of the hyaline cartilage gene collagen II, showed an upregulation tendency similar with collagen II, which reversely confirmed the reliability of the data. The increase in expression levels of aggrecan was highest in the 25 : 75 group, with 12.14 ± 4.26. In summary, when cocultured with pACs, collagen II, Sox9, and aggrecan were upregulated in hWJMSCs proportionally to the ratio of pACs; collagen I was also upregulated and collagen X was downregulated (Figure 4(a)).

Relative to the single pAC (0 : 100) group, the overall trend in gene expression levels of pACs in coculture groups showed that Sox9, aggrecan, collagen I, and collagen X were upregulated to different extents and collagen II was slightly downregulated. In the 75 : 25 group, collagen II was evidently downregulated, which reflected, to a degree, the dedifferentiation of pACs after coculture with hWJMSCs. Collagen I and collagen X were obviously upregulated, in the 75 : 25 group with 4.00 ± 1.00 and 4.70 ± 0.60, respectively, in the 50 : 50 group with 2.80 ± 0.70 and 8.00 ± 1.50, respectively. Aggrecan distinctly increased in the 75 : 25 group with 1.73 ± 0.35. In summary, after coculturing with hWJMSCs, the Sox9 and aggrecan levels of pACs were upregulated, while collagen II was downregulated. Collagen I and collagen X exhibited trends in different coculture groups (Figure 4(b)).

## 4. Discussion

Seed cells, mainly derived from MSCs and ACs, are a core element in articular cartilage tissue engineering. However, their study and applications are made difficult because (i) ACs are isolated from limited articular cartilage resources and easily dedifferentiate upon growth *in vitro* and (ii) MSCs maintain an unstable chondrogenic differentiation status. So, upon extensive induction, they can differentiate into bone tissue, or when using an insufficient amount of chondrogenic inducers, they do not properly differentiate into chondrocytes [34–36]. Here, disregarding the traditional seed-cell processing method, hWJMSCs were cocultured in a simulated articular cartilage microenvironment using pACs and an ACECM-oriented scaffold to construct the tissue engineering cartilage. From the *in vitro* results, during culturing on ACECM-oriented scaffold, hWJMSCs grew rapidly and seldom died. Besides, the 100 : 0 (hWJMSC) group also exhibited a more compact engineering tissue with a higher cell population and greater secretion of extracellular matrix compared with other groups. This demonstrated that ACECM has good biocompatibility with hWJMSCs. However, after coculturing with pACs and ACECM-oriented scaffold for 5 days, the proliferation rate of hWJMSCs evidently declined while that of pACs moderately sped up. This indicated that coculturing of pACs and hWJMSCs in the ACECM-oriented scaffold exerted a distinctly different influence on the proliferation of these two kinds of seed cells. We performed histological and immunohistochemical staining; although H&E staining showed an abundant extracellular matrix and many cells in the 100 : 0 group, safranin “O” and collagen II staining revealed that the coculture groups secreted amounts of glycosaminoglycan and collagen II at three weeks that were comparable with those of monoculture groups. Besides, the western blotting results also showed that the total protein amount of the cell scaffold complex was higher in the 25 : 75 and 50 : 50 groups compared with other groups. The advantages of coculturing with the cell scaffold complex were visible only after three weeks. The RT-PCR results at three weeks demonstrated that collagen II and aggrecan genes of hWJMSCs were upregulated with increasing percentages of pACs, whereas hWJMSCs, upon coculturing in the simulated in situ microenvironment, underwent differentiation into chondrocytes without any chondrogenic inducer. The microenvironment constructed by pACs and ACECM-oriented scaffold probably could induce hWJMSCs to differentiate into chondrocytes without the use of chondrogenic inducer. Some factors that may contribute to this observation are the following: (1) hWJMSCs derived from the umbilical cord Wharton's jelly had a better ability to differentiate into chondrocytes compared with adipose and bone [30, 37, 38]; (2) pACs possessed a primitive articular cartilage cell phenotype and could secrete some cytokines that were beneficial to chondrogenic differentiation of hWJMSCs. (3) The microenvironment constructed by pACs and the ACECM-oriented scaffold quite closely resembled the natural articular cartilage microenvironment, and the microenvironment has a huge effect on, and plays a critical guidance role in, the destiny of MSCs [19, 21]. In addition, at the same moment, collagen I and collagen II levels of pACs were upregulated and downregulated, respectively, in the coculture groups. A gene upstream of collagen II, Sox9 was upregulated in pACs in the coculture groups revealing a contradictory inverse correlation between collagen II and Sox9. However, some researchers reported that Sox9 gene also plays an important role in proliferation, which not only explains that RT-PCR results of pACs are plausible but also shows a possible reason why the proliferation of pACs was improved after coculture with hWJMSCs [39, 40].

However, this paper has left some questions unanswered and we will need to carry out more research to elucidate the functional mechanisms. For example, we have not identified the specific cytokines secreted by pACs and hWJMSCs and their signal pathways. It is important to further clarify the mechanism of hWJMSCs that has been induced to differentiate into chondrocytes by the microenvironment constructed by pACs and an ACECM-oriented scaffold which is very important to aid the development of and to optimize the coculture model to construct tissue engineering cartilage.

## 5. Conclusions

The engineered articular cartilage was successfully constructed using hWJMSCs cocultured with pACs in an ACECM-oriented scaffold. A potential mechanism behind the improved engineering cartilage tissue was (i) the primitive hyaline cartilage phenotype and the improved cytoactivity of pACs or (ii) the stable chondrogenic differentiation of hWJMSCs in a simulated in situ cartilage microenvironment constructed by pACs and the ACECM-oriented scaffold. There were some clear advantages: the repair and regeneration of the tissue were improved by the articular cartilage histochemical components and structural characteristics; pACs showed a native chondrocyte phenotype because they were not passaged *in vitro*; and hWJMSCs were induced to differentiate into chondrocytes by a simulated native cartilage microenvironment instead of inducers only and they replaced part of the pACs which to a certain extent released its resource limitation. It was a promising strategy that the simulated cartilage microenvironment constructed by pACs and the ACECM-oriented scaffold cocultured with hWJMSCs could effectively improve the quality of hyaline cartilage.

## Data Availability

The authors would like to share all the original data from our lab, including supplementary information that supports the findings of our manuscript, by depositing them in a publicly available data repository wherever possible. Usage restrictions, distribution, and reproduction of the original work provided via any medium are properly cited.

## Supplementary Materials

Supplementary Materials Table 1: primer sequences of goat target genes and human target genes for gene expression analysis.
