# Supplementary material for: Coculture of hWJMSCs and pACs in Oriented Scaffold Enhances Hyaline Cartilage Regeneration In Vitro
Source: Stem Cells Int. 2019 Feb 7;2019:5130152. doi: 10.1155/2019/5130152 (PMC6383394; doi:10.1155/2019/5130152)
Supplement: Supplementary Materials — Table 1: primer sequences of goat target genes and human target genes for gene expression analysis. [file 5130152.f1.docx]

Table 1. Primers sequences of goat target genes and human target genes for gene expression

Analysis.

| Target genes(goat) Primer sequences(5’-3’) |
| --- |
| Rbt GAPDH-F -CCTGGAGAAACCTGCCAAGT-  Rbt GAPDH-R -TGTCGCTGTTGAAGTCGCA-  Rbt Collagen1A1-F -AGACAGAGCAGAAACATCGGA-  Rbt Collagen1A1-R -CGGTGACACACAAAGACAAGA-  Rbt Collagen2A1-F -CCACACTCAAGTCCCTCAACA-  RbtCollagen2A1-R -AGTAGTCTCCGCTCTTCCACT-  Rbt Sox9-F -TCGCAGAAAGAACCCAAGAA-  Rbt Sox9-R2 -CAGTCTAACGACAAGGCGGT-  Rbt Collagen10-F -ATACCAAACACCTACGGGCA-  Rbt Collagen10-R -ACCCTGCTCTCCTCTTAGTGA-  Rbt Aggrecan-F -GTCCACCATTCGGCATAACC-  Rbt Aggrecan-R -ACTGAACTCTGGCAAACCCG- |
| Target genes(human) Primer sequences(5’-3’) |
| Rbt HGAPDH-F(Q) -CAAGAAGGTGGTGAAGCAGG-  Rbt HGAPDH-R(Q) -GGTGTCGCTGTTGAAGTCAG-  Rbt HCollagen1A1-F(Q) -GCCCTGTCTGCTTCCTGTAA-  Rbt HCollagen1A1-R(Q) -CAGTTTGGGTTGCTTGTCTGT-  Rbt HCollagen2A1-F(Q) -ACCCAGAAACAACACAATCCG-  RbtHCollagen2A1-R(Q) -GGTCAGGTCAGCCATTCAGT-  Rbt HSox9-F(Q) -TACACCGACCACAGAACTC-  Rbt HSox9-R(Q) -TAGACGGGTTGTTCCCAGTG-  Rbt HCollagen10A1-F(Q) -ACCTTGCTCTCCTTACTGC-  Rbt HCollagen10A1-R(Q) -ACCTTGCTCTCCTCTTACTGC-  Rbt HAggrecan-F(Q) -GCCAGCACCACCAATCTAAG-  Rbt HAggrecan-R(Q) -CAGTAACACCCTCCACGAACT- |
